# A Novel PPG-Based Biometric Authentication System Using a Hybrid CVT-ConvMixer Architecture with Dense and Self-Attention Layers

**DOI:** 10.3390/s24010015

**Published:** 2023-12-19

**Authors:** Mostafa E. A. Ibrahim, Qaisar Abbas, Yassine Daadaa, Alaa E. S. Ahmed

**Affiliations:** 1 College of Computer and Information Sciences, Imam Mohammad Ibn Saud Islamic University (IMSIU), Riyadh 11432, Saudi Arabia; meibrahim@imamu.edu.sa (M.E.A.I.); ymdaadaa@imamu.edu.sa (Y.D.); asmohamed@imamu.edu.sa (A.E.S.A.); 2 Department of Electrical Engineering, Benha Faculty of Engineering, Benha University, Benha 13518, Qalubia, Egypt; 3 Electrical Engineering Department, Faculty of Engineering at Shoubra, Benha University, Cairo 11629, Egypt

**Keywords:** biometric authentication, internet of things, photoplethysmography (PPG), deep learning, ConvMixer model, feature extraction, attention mechanisms, secure identification, authentication systems

## Abstract

Biometric authentication is a widely used method for verifying individuals’ identities using photoplethysmography (PPG) cardiac signals. The PPG signal is a non-invasive optical technique that measures the heart rate, which can vary from person to person. However, these signals can also be changed due to factors like stress, physical activity, illness, or medication. Ensuring the system can accurately identify and authenticate the user despite these variations is a significant challenge. To address these issues, the PPG signals were preprocessed and transformed into a 2-D image that visually represents the time-varying frequency content of multiple PPG signals from the same human using the scalogram technique. Afterward, the features fusion approach is developed by combining features from the hybrid convolution vision transformer (CVT) and convolutional mixer (ConvMixer), known as the CVT-ConvMixer classifier, and employing attention mechanisms for the classification of human identity. This hybrid model has the potential to provide more accurate and reliable authentication results in real-world scenarios. The sensitivity (SE), specificity (SP), F1-score, and area under the receiver operating curve (AUC) metrics are utilized to assess the model’s performance in accurately distinguishing genuine individuals. The results of extensive experiments on the three PPG datasets were calculated, and the proposed method achieved ACCs of 95%, SEs of 97%, SPs of 95%, and an AUC of 0.96, which indicate the effectiveness of the CVT-ConvMixer system. These results suggest that the proposed method performs well in accurately classifying or identifying patterns within the PPG signals to perform continuous human authentication.

## 1. Introduction

**Figure 1:**
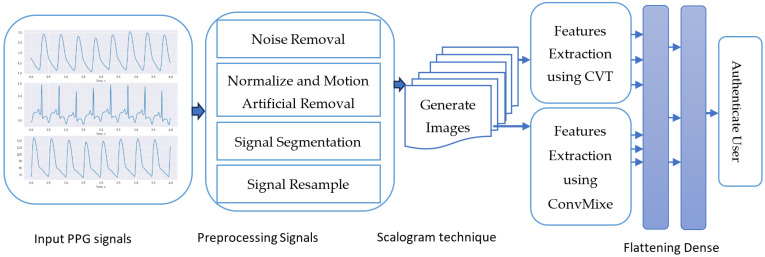
Structural outline diagram of the proposed CVT-ConvMixer with preprocessing PPG signals.

Biometric authentication is an essential aspect of identity verification systems, offering a secure and reliable means of confirming individuals’ identities [1]. Traditional biometric methods, such as fingerprint or iris recognition, have been widely adopted [2]. However, the integration of emerging technologies, such as Photoplethysmography (PPG) and deep learning (DL), presents exciting opportunities to enhance biometric authentication systems [3]. PPG is a non-invasive optical technique that measures blood volume changes in tissues. It involves the use of light sensors to capture the reflected light from the tissue, providing insights into cardiovascular activity and physiological characteristics. PPG signals can be obtained from various parts of the body, including the fingertip, wrist, or earlobe [4]. These signals exhibit unique patterns that are specific to individuals, making them suitable for biometric authentication [5], as shown in Figure 1.

Deep learning, a subset of machine learning, has gained significant attention due to its ability to learn complex patterns and representations from large amounts of data [6]. DL models, such as convolutional neural networks (CNNs) and recurrent neural networks (RNNs), excel at extracting meaningful features and making accurate predictions [7]. By leveraging DL techniques, biometric authentication systems can effectively analyze PPG signals and differentiate between individuals based on their unique physiological characteristics. The integration of PPG and DL for biometric authentication [8] offers several advantages. Firstly, PPG is a non-invasive and contactless method, making it more user-friendly compared to traditional biometric techniques. Additionally, DL models can learn intricate patterns and adapt to individual variations, enabling robust and accurate identification. Furthermore, PPG-based biometric authentication systems can potentially provide continuous and passive authentication, allowing for seamless user verification in various scenarios. However, there are challenges associated with this integration. Variability in PPG signals due to factors like skin tone, motion artifacts, and environmental conditions can impact system performance. Moreover, the collection and preprocessing of high-quality PPG data are critical for training reliable DL models. Addressing these challenges requires careful data selection, preprocessing techniques, and model optimization.

Several studies have investigated the use of CNNs to analyze PPG signals for biometric authentication. These CNN-based approaches typically involve preprocessing the PPG signals, extracting temporal features, and using CNN architectures to learn discriminative representations. The performance of CNN-based models is often evaluated based on metrics such as accuracy, sensitivity, specificity, and the area under the receiver operating characteristic (ROC) curve. RNNs, especially variants like long short-term memory (LSTM) and/or gated recurrent units (GRU), have also been explored for biometric authentication using PPG signals. RNNs are well-suited for capturing temporal dependencies in sequential data, making them suitable for analyzing PPG signals, which exhibit temporal characteristics. These models can learn long-term dependencies and capture patterns that are crucial for user identification.

More recently, transformer-based models, originally designed for natural language processing tasks, have shown promise in analyzing PPG signals for biometric authentication. Transformers leverage self-attention mechanisms to capture global dependencies in the data and have demonstrated strong performance in various domains. These models can capture long-range dependencies in PPG signals and have the potential to improve authentication accuracy. Additionally, there has been research on combining PPG signals with other modalities, such as ECG or accelerometer data, to enhance the performance and robustness of biometric authentication systems. Multimodal fusion approaches aim to leverage complementary information from different modalities to improve identification accuracy and address challenges such as noise and variability in PPG signals.

This paper aims to explore the integration of PPG and advanced DL for biometric authentication. It presents different steps of the methodology, including data collection, preprocessing, feature extraction, DL model training, and validation. The challenges involved in this process are discussed, along with potential solutions. The results of PPG-based biometric authentication systems using DL techniques are presented, showcasing their potential and effectiveness.

### 1.1. Major Contributions

The system developed in this paper contributes significantly to the field of biometric authentication, particularly in the context of Photoplethysmography (PPG) and deep learning (DL) techniques. Biometric authentication is a crucial area for verifying individual identities, and the integration of preprocessing and signal transformation in PPG and DL methods holds immense promise for enhancing its effectiveness. We have made the following contributions:

1. A novel scalogram-based and hybrid CVT-ConvMixer model is being developed to identify humans using preprocessed PPG signals.
2. The research introduces a novel approach using Scalograms, a 2-D image technique, to enhance PPG signal analysis and strengthen the reliability of biometric authentication systems amidst signal variability.
3. A biometric authentication system utilizes the CVT-ConvMixer hybrid deep learning model, enhancing robustness and performance by effectively managing signal variations in PPG data for accurate individual identification.
4. Attention mechanisms enhance the ConvMixer model, capturing key temporal PPG signal features and improving individual differentiation in biometric authentication. Extensive evaluations of various datasets and performance metrics underscore its effectiveness and accuracy in identifying authentic users.
5. Experiments on three PPG datasets highlight the developed approach’s effectiveness in accurately classifying patterns, ensuring reliable continuous authentication of individuals through high recognition rates.

Finally, this research contributes significantly to the field of biometric authentication by addressing the challenges posed by the variability of PPG signals. It introduces a novel Scalogram-based representation and a powerful CVT-ConvMixer hybrid classifier with attention mechanisms. The high recognition rates achieved in real-world scenarios underscore the practical implications and potential impact of this work in enhancing the security and reliability of biometric authentication systems.

### 1.2. Article Outline

The following structure is adopted for the remainder of this paper: Section 2 describes the literature review of previous studies. Section 3 provides a detailed account of the methods and materials employed in the proposed research, encompassing PPG signal acquisition, signal transformation, feature extraction, and classification. In Section 4, the experimental results are performed and compared. Section 5 presents the discussions, advantages, and future work of the proposed system. Lastly, Section 6 concludes this paper.

## 2. Related Works

Biometric authentication using PPG signals [9] has gained significant attention in recent years. PPG signals, which capture the volumetric changes in blood vessels, provide unique physiological information that can be used for user identification and authentication. Deep learning techniques, including convolutional neural networks (CNNs), recurrent neural networks (RNNs), and transformer-based models, have been applied to extract features and patterns from PPG signals.

Overall, the literature highlights the potential of deep learning techniques for biometric authentication using PPG signals. However, it is important to consider the specific characteristics of the dataset, preprocessing techniques, model architectures, and evaluation metrics when implementing and comparing different approaches. Conducting a thorough literature review will provide more in-depth insights and help identify the most relevant and effective techniques for a specific research or application. The integration of Photoplethysmography (PPG) and deep learning (DL) techniques for biometric authentication has gained increasing attention in recent years. Several studies have explored this intersection, examining PPG-based biometric authentication’s feasibility, accuracy, and effectiveness using DL models. This literature review provides an overview of key research in this area.

One study in [10] proposed a PPG-based biometric authentication system using a combination of DL and traditional machine learning techniques. Their work demonstrated the potential of DL models, specifically CNN, for accurately identifying individuals based on PPG signals acquired from the fingertip. The study highlighted the importance of feature extraction and preprocessing methods to improve system performance. This study explores the use of deep learning techniques for biometric identification using PPG signals. The authors propose a deep learning framework that integrates convolutional and recurrent neural networks to extract features from PPG signals and classify individuals. The results demonstrate the effectiveness of the proposed method, achieving high accuracy in biometric identification tasks.

In another research effort [11], the authors explored the use of recurrent neural networks (RNNs) for PPG-based biometric authentication. They collected PPG signals from the wrist and developed a two-layer CNN with an RNN model to learn the temporal dynamics of the signals. The study achieved promising results, indicating the suitability of CNN-RNNs for capturing sequential information and enhancing authentication accuracy. There are 22 PPG recordings in the TROIKA dataset that were collected during diverse physical activities. On 20 subjects, the authors attained a mean absolute heart rate (HR) estimation error of 1.47, 3.37 beats per minute, and an average accuracy of 96%. Overall, the proposed approach aims to leverage ECG signals and ensemble Siamese Network (ESN) techniques to develop an authentication system with high accuracy and robustness [12]. The authors used the ECG-ID and PTB datasets and reported accuracy of 93.6% and 96.8%, respectively.

A new large-scale PPG dataset known as PPG-DaLiA that enhances PPG-based continuous heart rate monitoring methods was developed in a study [13]. Through extending a state-of-the-art algorithm and incorporating DL with various CNN architectures, it addresses challenges like motion artifact compensation and scenario-specific optimizations of prior methods. A CNN model is presented in the study of [14] to identify unique and time-stable features in PPG data, employing two layers with convolutional kernels, SELU, and dropout, followed by a fully connected layer utilizing sigmoid and binary cross entropy for classification. Incorporating strategies from prior studies, L2 regularization, and 10-fold cross-validation prevent overfitting, while the ADAM optimizer with a 0.0001 learning rate fine-tunes the model over 60 epochs. The experiments were performed on four different datasets, such as PRRB, TROIKA, Biosec1, and Biosec2. The variation-stable approach in the study of [15] utilizes four score fusion techniques, aiming to identify unique, time-stable features in PPG data, showcasing superior verification system performance across various datasets and sessions.

In another study [16], the authors developed a Homomorphic random forest (HRF) to classify homomorphically encrypted biometric features, ensuring that the user’s PPG biometrics remain protected during the authentication process. Additionally, we have implemented beat qualification screening to filter out unqualified signals, and from the 541 extracted features, 19 features were selected with minimal redundancy as the user’s biometric features. This selection process ensures authentication accuracy, as demonstrated in our evaluations using five PPG databases collected through three different collection methods (contact, remote, and monitor). Notably, they performed experiments to incorporate PPG signals acquired from remote cameras. The experimental results highlight that our biometric system achieves an average accuracy of 96.4%, an F1 score of 96.1%, an Equal Error Rate (EER) of 2.14%, and an authentication time of approximately 0.5 s.

The authors of [17] introduced a biometric identification model employing a one-dimensional Siamese network with photoplethysmogram (PPG) signals. In the preprocessing phase, the authors intend to preserve the unique characteristics of each individual while reducing noise. Instead of using bandpass or low-pass filters, a method of multicycle averaging was employed. They examined the efficacy of this procedure by varying the number of cycles and comparing the outcomes. Authentic and counterfeit data are utilized for biometric identification verification. The one-dimensional Siamese network is used to measure class similarity and discover that the procedure with five overlapping cycles produces the most promising results. Excellent identification performance is observed using overlapping data from five single-cycle signals, with an AUC score of 0.98 and an accuracy of 0.9723. 

PPG biometrics have garnered considerable attention [18], and while DL has demonstrated optimistic results, this discipline still faces a number of obstacles. To address these obstacles, the authors developed a novel method for PPG biometric recognition dubbed the Dual-Domain and Multiscale Fusion Deep Neural Network (DMFDNN). Their model’s 5-fold cross-validation accuracy on the Structural Performance Category (SPC) dataset was 96%. In contrast, the performance in terms of F1 score (72%) and precision (67%) was significantly inferior. In their study [19], the authors proposed using PPG as a biometric indicator by employing end-to-end learning with an XgBoost classifier. During the period when PPG signals are acquired, unique physiological factors were considered in the selection of features. As a classification model, they adopted the XgBoost algorithm. The authors achieved an ACC of 97.36%. Their method obtained an AUC of 83 and an average accuracy of 78.2%.

The study in [20] proposes an end-to-end architecture using Convolutional Networks for biometric authentication via PPG biosensors, tested on two databases: Troika and PulseID. Achieving an AUC of 78.2% and 83.2% on PulseID and Troika, respectively, the approach validates the efficacy of using raw PPG signals for authentication in e-health and fitness contexts, demonstrating promising biometric potential. Despite the notable success and low input throughput, further research is necessary to address and comprehend the variability in certain subjects. The authors in [21] presented a biometric authentication method utilizing PPG signals. They applied a deep learning model combining CNN and LSTM to PPG signals, achieving an average accuracy of 87.1%. However, this method had limitations in terms of long inference time due to a high number of parameters and model complexity. Whereas in the study of [22], the authors developed a low-cost continuous system utilizing wrist-worn photoplethysmogram (PPG) sensor pulse signals. Experimental results with 20 participants using a wrist-worn PPG detection platform demonstrated that their system achieved a high classification accuracy (CA) of over 90% and a low false-positive rate of 4% when detecting random attacks. Similar to this study, the previous research focused on effortless continuous authentication using PPG signals. However, their approach utilized the entire signal by correcting it with MA, whereas our study only employed high-quality signals for authentication, excluding false signals caused by noise. 

In the study of [23], the authors presented a biometric system that utilizes plethysmography (PPG) signals and employs a fuzzy min-max neural network for recognition purposes. PPG signals offer the advantage of liveness detection, distinguishing them from other biometric methods such as fingerprint recognition and face recognition. The proposed system comprises three main processes: signal preprocessing, feature extraction, and model classification. Signal preprocessing ensures the quality of PPG signals, while feature extraction captures relevant information for recognition. Experimental results obtained from the Capnobase database demonstrate the effectiveness of the system, achieving an accuracy rate of 97.62%. 

This work introduces a novel security management approach for authentication [24] that utilizes two combined convolutional neural networks (CNNs) for the biometric identification of users. The approach incorporates Federated Learning (FL), a machine learning paradigm that enables collaborative training of decentralized models without sharing sensitive data, thus ensuring data privacy and management. In their study of [25], the authors developed a new session-based continuous authentication scheme utilizing photoplethysmography (PPG) signals. This model consists of two phases, such as feature extraction using deep autoencoders from PPG signals and authentication using the Local Outlier Factor (LOF). Despite its simplicity, the proposed solution achieves remarkable performance, with an F1 score of 91.3% on the CapnoBase dataset and 91.0% on the BIMDC benchmarking dataset. User authentication (UA) is an essential process that utilizes biometric techniques [26] to grant individuals access to physical or virtual spaces. The authors used PPG signals as an optical sensing method. Experimental results demonstrate that our Bi-LSTM-based UA algorithm, employing both feature-based machine learning and raw data-based deep learning approaches, achieves accuracy rates of 95.0% and 96.7%, respectively.

A hardware prototype for the authentication of real-time training data [27] has been developed with success. According to reports, the equal-error rate at the authentication stage is 5.5%, while the identification accuracy reaches 98%. These results demonstrate that the proposed system achieves accurate and trustworthy user authentication. The healthcare industry is embracing personalized smart care models enabled by the Internet of Things (IoT) and Artificial Intelligence (AI) [28]. The proposed model is evaluated using physiological biometric characteristics, namely Electrocardiogram (ECG) and photoplethysmography (PPG) signals, obtained from five publicly accessible datasets in repositories such as Physionet and Mendeley. Multiple AI models are trained and evaluated, and the multimodal fusion authentication model demonstrates promising results with an accuracy of 99.8 percent and an Equal Error Rate (EER) of 0.16 percent. These results demonstrate the effectiveness of the proposed model in enhancing the accuracy and security of authentication in healthcare scenarios. In [29], the system was evaluated using a dataset containing samples from 42 individuals. It was determined that the average classification accuracy for identity verification was 99.16%, with a false match rate (FMR) of 0.56% and a false non-match rate (FNMR) of 13.50%. The error in rank-1 identification was 7.24 percent. These results, derived from the dataset under consideration, either surpass or are comparable to the performance of the best-performing methods described in the extant literature.

Overall, the literature assessment reveals that the integration of PPG and deep learning (DL) techniques offers promising avenues for biometric authentication. DL models, combined with PPG signals from various body parts or remote sensing, have shown effective identification capabilities. Signal quality assessment and preprocessing techniques are crucial for ensuring the reliability of PPG-based biometric authentication systems [30]. Further research is needed to address challenges related to individual variability, environmental factors, and generalizations to real-world scenarios. PPG data acquisition involves the collection of Photoplethysmography (PPG) signals from individuals for further analysis and processing in biometric authentication systems.

## 3. Methodology

### 3.1. Data Acquisition

The Real-World PPG datasets [31,32,33], publicly available, were utilized to evaluate the proposed preprocessing steps and CVT-ConvMixer model. The dataset comprises PPG signals obtained under uncontrolled conditions. It consists of PPG signals recorded from 35 individuals, with each signal captured for a duration of 5 s at a sampling rate of 50 samples per second. The dataset encompasses 2074 data points, of which 1374 (approximately 66%) were allocated for training, while the remaining 700 (approximately 34%) were designated as the test set. To mix these three datasets, we used a cross-validation test. Notably, the PPG signals within the Real-World PPG (RW-PPG) dataset [31] are characterized by low noise. This study utilized the dataset to mitigate the impact of disturbances such as motion artifacts and to explore the potential of PPG waveform characteristics.

In addition, we have used the Beth Israel Deaconess Medical Center (BIDMC) [32] and Multiparameter Intelligent Monitoring for Intensive Care (MIMIC) [33]. The BIDMC dataset comprises 8 min recordings of PPG signals (sampling frequency, fs = 125 Hz) acquired from adult patients. The MIMIC database collects recordings of patients and is published on PhysioBank for free. Table 1 describes the details of the three datasets used in this paper to evaluate the performance of the CVT-ConvMixer model.

### 3.2. Proposed Methodology

The following phases have been developed to integrate PPG and deep learning (DL) for biometric authentication, as shown in Figure 1. This research presents a pioneering methodology that significantly advances the field of biometric authentication, particularly in the context of Photoplethysmography (PPG) and deep learning (DL) techniques. It introduces a novel representation called a Scalogram, which visually captures time-varying frequency components of multiple PPG signal segments. This innovative approach enhances the system’s robustness and reliability. A biometric authentication system is developed using deep learning techniques, specifically the CVT-ConvMixer hybrid classifier. This model combines the strengths of both architectures, improving feature representation and information extraction. It excels in real-world scenarios, accurately identifying individuals despite signal variations. Attention mechanisms are integrated into the ConvMixer model to capture temporal relationships and informative features within PPG signals. This enhancement significantly boosts the model’s ability to distinguish genuine users. In practice, this research introduces innovative techniques for handling PPG signal variability, harnesses the power of deep learning for biometric authentication, and achieves high accuracy in identifying individuals, showcasing its potential for real-world applications in security and authentication. These steps are described in the subsequent paragraphs.

#### 3.2.1. Preprocessing

**Figure 2:**
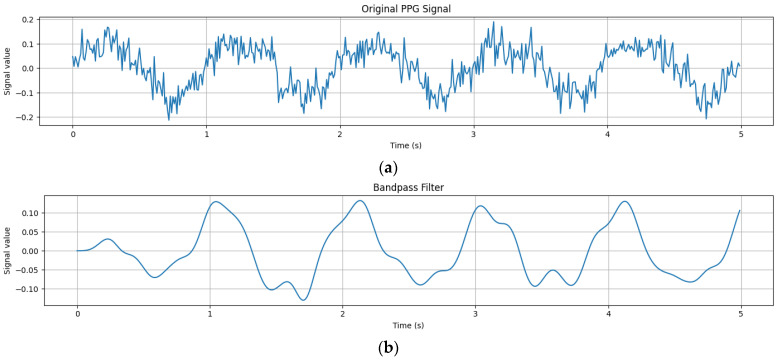

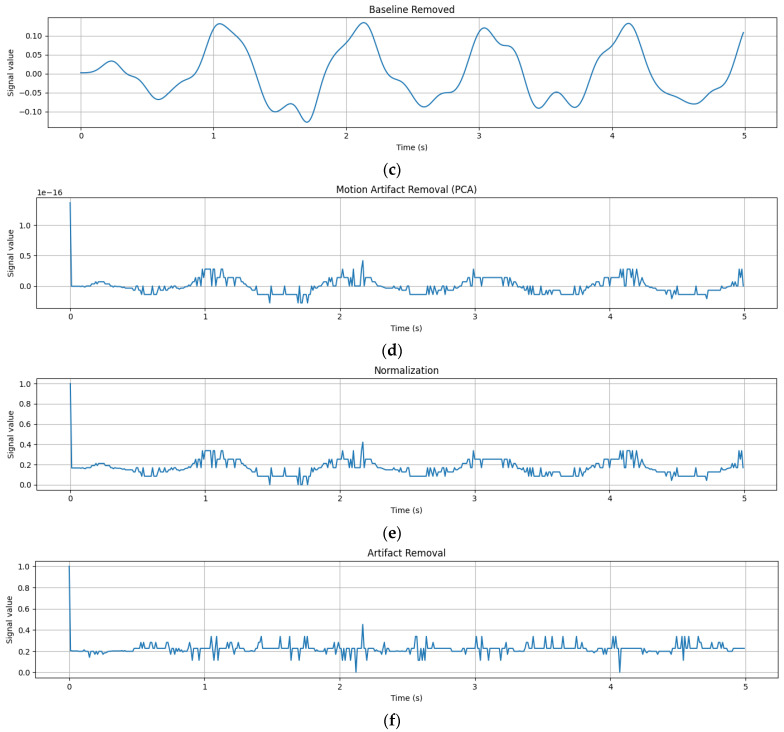
An example of a visual representation of preprocessed PPG signals is (**a**) the original input of PPG signals; (**b**) the application of a bandpass filter; (**c**) baseline removal; (**d**) motion artifact removal by PCA; (**e**) signal normalization and resampling; and (**f**) artifact rejection.

Preprocessing the photoplethysmography (PPG) signals is an essential step in analyzing and extracting heart biometric information. Figure 2 visually illustrates the preprocessing of PPG signals. While the preprocessing steps are presented in Algorithm 1, PPG signals are crucial for capturing volumetric changes in blood vessels, offering insightful data about the cardiac cycle, heart rate, and various cardiovascular parameters. In preprocessing these signals for authentication systems, overlapping segments are typically employed to ensure no vital transitional information is missed between segments. A common practice is to use an overlap of 50%. For example, with 5 s segments, the subsequent segment would indeed start 2.5 s into the current one. This overlapping technique is essential for capturing more robust features in the PPG signal and is particularly beneficial for detecting short-lived but significant events or artifacts.

First step: if we are monitoring the PPG signal of a person to extract information about their heart rate and cardiovascular health, then we must perform preprocessing on the PPG signals. Initially, a raw PPG signal that contains noise from various sources, including ambient light fluctuations and electrical interference, is captured. To clean up the signal, we applied a bandpass filter. This filter removes high-frequency noise (like rapid fluctuations caused by ambient light) with a low-pass filter and eliminates baseline wander (slow drifts caused by motion or pressure changes) with a high-pass filter. The resulting signal is now smoother and focused on the pulsatile component, making it easier to analyze. Despite filtering, the PPG signal might still have some residual baseline drift due to small movements or changes in pressure. We have subtracted this baseline from the signal to isolate the variations caused by the pulsatile blood flow. Subtracting the baseline from a PPG signal isolates variations caused by pulsatile blood flow, enabling precise analysis of cardiovascular conditions and heart rate by minimizing interferences from non-pulsatile components. This step ensures that the data are centered around zero and helps in accurate heart rate extraction. During the recording, the person might move, leading to motion artifacts in the PPG signal. To address this, we have applied the motion artifact removal technique, principal component analysis (PCA). The idea is that the principal component(s) that capture the most variance in the signal may represent the true PPG signal, while the components capturing less variance might represent noise or artifacts. In practice, PCA is a linear method that is used for motion artifact removal in PPG signals due to its simplicity, computational efficiency, effectiveness in isolating vital signals, and capacity for reducing noise and dimensionality, making it broadly applicable.

To ensure consistency when comparing PPG signals across different individuals or sessions, we implemented a normalization process. This process involves scaling the signal’s amplitude, but rather than simply dividing the samples by the value of the highest amplitude peak, we chose a more tailored approach. We adjusted the signal so that its peak amplitude aligns with a specific physiological parameter, like blood volume change. This method of normalization, as opposed to restricting values to the [0, 1] interval, allows for a more physiologically relevant comparison of signals. It ensures that, despite varying signal amplitudes from different sources, the comparison focuses on meaningful physiological variations rather than just numerical values. As mentioned earlier in Section 3.1, we have collected PPG signals from different sources, such as RW-PPG, BIDMC, and MIMIC. Therefore, it is very important to apply this normalization step to the collected datasets.

Given the variability in sampling rates of PPG signals, which can differ based on the recording equipment or conditions, we standardized these signals by resampling them at a uniform rate. For instance, since the original signals had sampling rates of 250 Hz and 500 Hz, we resampled all signals to a common rate of 250 Hz using a bicubic interpolation method. This resampling step is vital for ensuring accurate feature extraction and consistent analysis across all signals. After this preprocessing step, we proceed to assess the quality of the PPG signal, ensuring that the data used in our analysis maintains a high standard of consistency and reliability.

The PPG signal quality is evaluated by identifying segments where the signal quality might be compromised. We used instances where the signal-to-noise ratio (SNR) is low, indicating that the desired signal is being obscured or contaminated by noise. This suggests a process wherein the pre-processed PPG signals are scrutinized for segments where the reliability or clarity of the signal might be compromised due to external factors or inherent noise, ensuring that subsequent analyses or feature extraction are based on high-quality, reliable data. These assessments would likely involve analyzing the consistency, stability, and clarity of the signal waveform across the recorded data.
**Algorithm 1:** Preprocessing PPG Signals to Remove Noise and Artifacts
  
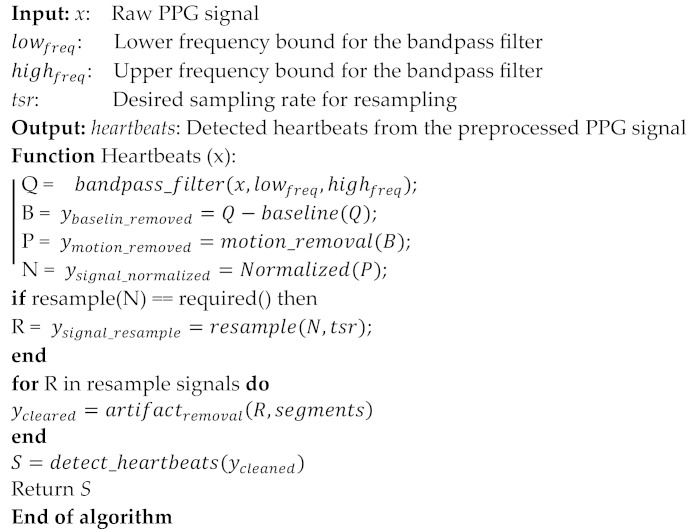


#### 3.2.2. Signal Transformation

Converting preprocessed PPG signals into 2-D images is a technique commonly used for further analysis. The overall steps are described in Algorithm 2. This algorithm is designed to transform multiple segments of preprocessed Photoplethysmography (PPG) signals into a 2D image known as a Scalogram. PPG signals are used to monitor various cardiovascular parameters, and Scalograms help visualize their frequency components over time.

The Continuous Wavelet Transform (CWT) is a technique in signal processing that differs from the Short-time Fourier Transform (STFT) by employing a dynamic window known as the main wavelet. This wavelet is both scaled and shifted during the transformation process, allowing for long time intervals at low frequencies and short time intervals at high frequencies. Unlike the STFT, where window sizes remain constant, the CWT can adapt by using windows of varying sizes, enabling it to effectively analyze both high- and low-frequency components in a time series [34]. This adaptability makes CWT particularly useful for analyzing non-stationary signals like EEG. The method utilizes smaller scales for high frequencies and larger scales for low frequencies to achieve optimal resolution. In practice, the choice between CWT and STFT depends on the specific characteristics of the signal and the analysis goals. CWT is advantageous when high adaptability, precise frequency localization, and detailed time-frequency information are required. On the other hand, STFT is computationally more efficient and may be suitable for simpler cases where fine-grained time-frequency analysis is not critical.

Mathematically, the continuous-time representation of CWT is defined by Equation (1):

(1) $$W_{x}\left(s,\tau \right)=\frac{1}{\sqrt{s}}\int_{-\infty }^{\infty }x(t)\psi^{’}\left(\frac{t-\tau }{s}\right)dt$$

- W(s, τ): are the wavelet coefficients. They represent the result of the CWT applied to a time signal *x*(*t*) at different scales (s) and positions (τ).
- *x*(*t*): is the time-domain signal to be analyzed using CWT. It could be any non-stationary signal, like an EEG signal.
- ψ(t): is the basic wavelet function and its complex conjugate. The choice of the wavelet function is crucial in CWT. We used the Morlet wavelet, which is known for its suitability in spectral analysis of non-stationary signals and is often preferred for its balance between time and frequency localization.
- s: is the scale parameter. It controls the width of the wavelet function in the time-frequency domain. The s parameter in wavelet transforms indicates that a scale factor greater than 1 is typically used to capture low-frequency components of a signal, while a scale factor less than 1 is used for high-frequency components.
- τ: is the position parameter, often referred to as time or shift. It determines where the wavelet function is centered in the time domain. Shifting the wavelet across the signal at different positions τ allows analyzing the signal’s time evolution at various points.

Continuous Wavelet Transform (CWT) is utilized to analyze PPG data and produce a visualization known as a scalogram. The PPG data, presumably a continuous-time signal, undergoes the CWT process, wherein it is analyzed through various frequency components by applying multiple expansions and time offsets of a wavelet, specifically the Morlet Continuous Wavelet in this context. The CWT allows the identification and visualization of local time-frequency energy density within the signal, depicted as a scalogram. Each segment of the PPG data are independently transformed through CWT, generating a scalogram image per segment. In this paper, we used a total of 500 images being generated, with 100 for each of the segments, indicating that the data are divided into distinct portions for analysis. These scalogram images effectively act as visual representations of the different frequency components present within each respective PPG signal segment, providing insight into the temporal and frequency characteristics of the blood volume changes during the cardiac cycles represented in each segment. Lastly, it is mentioned that sample scalogram images, likely exemplifying the resultant data post-CWT transformation, are displayed for three different individuals, potentially highlighting variations or unique features within their respective PPG signals.

We utilized the Morlet wavelet as a continuous wavelet transform (CWT) function as a fundamental step to develop a Scalogram-based image. Next, the algorithm expects a list of preprocessed PPG signal segments, which represent different time intervals of PPG data. Each segment is represented with values representing signal amplitudes at discrete time points. The algorithm proceeds by initializing an empty 2-D NumPy array called “image_matrix”. This array will store the Scalogram information for each PPG segment, with each row corresponding to a different segment and columns representing either time or frequency bins, depending on the Scalogram representation.

For each PPG segment in the input list, the algorithm performs the following steps: (1) Calculates the number of data points in the segment based on the specified as a segment_duration and sampling_rate. (2) Pad the PPG segment with zeros if its length is shorter than the desired length to ensure uniformity. (3) Utilizes the Continuous Wavelet Transform (CWT) to compute the Scalogram of the PPG segment. The chosen wavelet type, specified by the Morelet wavelet, affects the transformation. (4) Focuses on the magnitude of the frequency components by taking the absolute value of the Scalogram. (5) Resizes the Scalogram to match the desired image length specified by the desired_length parameter. The algorithm generates a 2-D image (a Scalogram) that visually represents the frequency content of multiple PPG signal segments using the Scalogram technique. This enables the analysis of time-varying frequency components in the PPG data. Users can modify parameters and replace, for example, PPG segments with their own data for specific applications.

**Figure 3:**
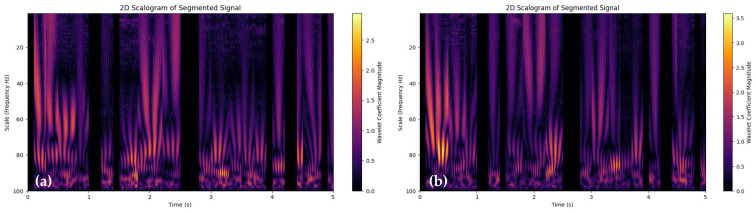
Visual representation of the frequency content of multiple PPG signal segments using the Scalogram technique for (**a**) person 1 and (**b**) person 2.

Regarding the frequency scale of the scalograms ranging from 0 to 100 Hz, as shown in Figure 3, it is important to clarify that while the standard heart rate range typically lies between 0.7 and 1.8 Hz, the wider frequency range in the scalograms is employed to capture not only the fundamental heart rate frequencies but also higher-frequency harmonics and transient events. These additional frequency components can provide valuable biometric information, such as subtle variations in heart rate patterns, which might be indicative of specific physiological or pathological conditions. The inclusion of a broader frequency range allows for a more comprehensive analysis of the PPG signal, which is crucial for the enhanced accuracy and reliability of our biometric authentication system.

**Algorithm 2:** Generating a 2-D Image (Scalogram) from Preprocessed PPG Signals
  

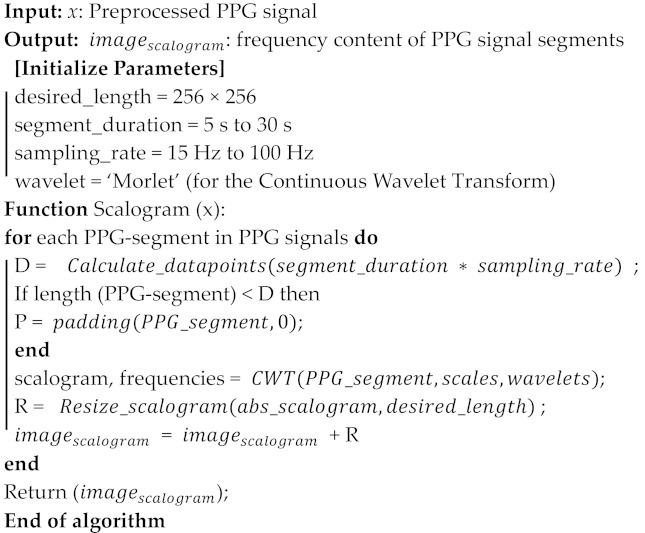



#### 3.2.3. Model Architecture–CVT-ConvMixer

This algorithm outlines the step-by-step process of building a hybrid model that combines the strengths of the Convolutional Vision Transformer and ConvMixer architectures. The resulting hybrid model is aimed at effectively classifying PPG signal feature vectors, ultimately contributing to biometric authentication efforts.

The purpose of this algorithm is to develop and train a hybrid model, combining the Convolutional Vision Transformer (CVT) and ConvMixer architectures, for classifying PPG signal feature vectors in the context of biometric authentication. The algorithm takes PPG signal feature vectors for training and testing, along with corresponding labels. First, the algorithm applies a preprocessing function to each PPG signal feature vector in the training dataset. This step may involve removing artifacts, normalizing data, and preparing it for further processing. The preprocessed signals are segmented into fixed-length segments. This segmentation process ensures that the signals are divided into smaller, manageable portions that can be fed into the model. To feed the segmented data into the model, the algorithm creates 2-D image maps from the segments. This involves reshaping the segments into a suitable image-like format. Zero-padding may also be applied to ensure consistent dimensions.

Convolutional Vision Transformer (CVT): The algorithm proceeds with the CVT component. It applies a Conv2D layer to generate convolutional embeddings from the image maps. The embeddings are then reshaped, and positional information is added to capture spatial relationships. Multi-head self-attention is applied using the MultiHeadAttention layer with parameters such as the number of heads and key dimensions. The attention outputs are aggregated to obtain CVT features [35], capturing relevant spatial information.

In our research, we introduce a novel concept called the Convolutional Transformer Block, which incorporates convolutional layers into the Transformer structure. We believe that strategically adding convolutions to the ViT (Vision Transformer) can enhance its performance and robustness while still maintaining computational and memory efficiency. To validate our idea, we propose the Convolutional Vision Transformer (CVT), which seamlessly integrates convolutions into the Transformer framework, ensuring efficiency in terms of parameters and floating-point operations (FLOPs). The CVT model comprises two key components: the Vision Transformer (VT) and Feature Learning (FLs). The FL component is responsible for extracting useful features from the Continuous Wavelet Transform-based Spectrogram (CWTS) images. These learned features are then passed to the VT component, which further transforms them into a series of image pixels for the final detection phase. Unlike the Inception v3 model, the FL component does not include a fully connected layer and is focused on extracting CWTS features rather than classification, effectively creating a CNN (Convolutional Neural Network) without the fully connected layer.

Within the FLs component, there are 17 convolutional layers, each using a 3 × 3 kernel size, employing ReLU activation for non-linearity, and incorporating batch normalization (BN) for normalizing output features. Max-pooling is applied five times with a 2 × 2-pixel window and a stride of 2, reducing the image dimensions after each pooling operation.

The VT component of the CVT model takes the feature map of the CWTS spectrogram as input, creating seven patches from the feature maps. These patches are then linearly embedded into a sequence of length 1 × 1024, and their positional information is retained by adding position embeddings (2 × 1024 dimensions). These embeddings are then sent to the Transformer.

The VT encoder in the Vision Transformer employs Multi-Head Self-Attention (MSA) and Multi-Layer Perceptron (MLP) blocks. The MLP block serves as a Feedforward Network (FFN), and the Transformer’s internal layers are normalized. The Transformer uses eight attention heads, and the MLP head consists of ReLU nonlinearity followed by two linear layers, akin to a fully connected layer in a typical CNN architecture. The first layer has 2048 channels, while the last layer has two channels. The CVT model consists of 38.6 million trainable parameters and a total of 20 weighted layers. For the final detection task, Softmax is applied to the MLP head’s output to obtain class probabilities.

ConvMixer Model: Next, the ConvMixer component is applied. Similar to CVT, the algorithm utilizes a Conv2D layer to create ConvMixer embeddings. Multi-head self-attention is then applied, followed by the aggregation of attention outputs to derive ConvMixer features. This captures relevant spatial information similar to CVT but with a different approach. This patch-based processing is a fundamental aspect of ConvMixer’s design [36], enabling the model to capture fine-grained features and achieve impressive performance on various tasks without the need for complex recurrent or attention mechanisms. 

The ConvMixer component is a novel architectural approach to deep learning, particularly suited for tasks requiring spatial understanding and feature extraction from images. It introduces a unique combination of Conv2D layers and multi-head self-attention mechanisms. Similar to traditional Convolutional Neural Networks (CNNs), ConvMixer uses Conv2D layers as its foundational building blocks. These layers perform convolution operations on the input data, applying learnable filters to extract local features. Conv2D layers have been widely successful in image processing tasks, allowing models to capture hierarchical features from low-level edges to high-level object representations.

To develop the CVT-ConvMixer model, we address the challenge of capturing long-term dependencies in low-frequency heart rate data by transforming this data into a 2D spatial representation, such as scalograms. This transformation enables the model to interpret heart rate signals not just over time but also across various frequency components, facilitating the detection of extended dependencies. Additionally, the integration of self-attention mechanisms within the model significantly enhances its capability to identify and prioritize the most informative segments of the data. These self-attention layers enable the model to focus on crucial parts of the signal, regardless of their position in the sequence. Consequently, this innovative combination of 2D signal transformation and self-attention allows our model to effectively recognize and utilize long-term patterns and dependencies, crucial for robust biometric authentication based on heart rate data.

After the initial Conv2D layers, ConvMixer incorporates multi-head self-attention mechanisms. Self-attention enables the model to weigh the importance of different spatial positions when processing information. We have selected the task-specific criteria for the ConvMixer model, which might learn to prioritize or weigh attention towards elements that are crucial for predictive accuracy based on the learned correlations from the training data. Multi-head attention refers to running multiple parallel attention mechanisms, each focusing on different parts of the input. This allows the model to capture long-range dependencies and relationships between various spatial positions in the image. The outputs of the multiple attention heads are then aggregated to form ConvMixer features. This aggregation step combines the information learned by different heads, effectively capturing a holistic understanding of the spatial relationships within the input data. This process is crucial for enabling ConvMixer to handle complex visual patterns and relationships effectively.

One of the distinctive features of ConvMixer is its reliance on patch-based processing. Instead of using complex recurrent or attention mechanisms to model spatial relationships, ConvMixer divides the input image into smaller patches and processes each patch independently. This patch-based approach allows ConvMixer to capture fine-grained details and spatial relationships without the computational complexity associated with sequential or attention-based processing. It also enables ConvMixer to achieve remarkable performance on a wide range of computer vision tasks.

ConvMixer is a powerful architectural innovation that combines Conv2D layers and multi-head self-attention to capture spatial information effectively. Its patch-based processing approach simplifies the modeling of spatial relationships, making it efficient and suitable for various computer vision tasks. The initial layer of ConvMixer applies these principles to start the feature extraction process. Therefore, the first layer of ConvMixer is calculated by Equation (2) as:

(2) $$Z_{0}=BNorm\left(\sigma Convol\to h\left(X,stride=p,\;kernelsize=p\right)\right)$$

The model’s second component is the primary ConvMixer layer, which is replicated for a certain depth. Within this layer, there is a residual block incorporating a depthwise convolution. In essence, a residual block combines the output of a prior layer with the output of a subsequent layer. In this specific scenario, the inputs are fused with the outcome of the depthwise convolution layer. Next to this fusion, there is an activation block, followed by a pointwise convolution, and another subsequent activation block, which is calculated by Equations (3) and (4) as:

(3) $$Z_{l}=BNorm\left(\sigma ConvolDepthwise\left(Z_{l-1}\right)\right)+Z_{l-1}$$

(4) $$Z_{l+1}=BNorm\left(\sigma Convolpointwise(Z_{l})\;\right)$$

The third component of the ConvMixer model introduces a crucial step: the inclusion of a global pooling layer. This step aims to derive a feature vector of size ‘h’ from the processed patches. Global pooling serves to compress the spatial dimensions of each patch into a fixed size, a pivotal transformation for subsequent tasks such as classification using a SoftMax classifier. In terms of activation functions, ConvMixer adopts the Gaussian error linear unit (GELU). GELU is a sophisticated and differentiable activation function renowned for its strong performance in deep neural networks. Unlike the Rectified Linear Unit (ReLU), which simply sets all negative values to zero, GELU applies a more intricate approach. It assigns weights to inputs based on their magnitude rather than solely relying on their sign, thus offering a more nuanced gating mechanism. This distinctive characteristic of GELU ensures the preservation of both positive and negative information within the activation, rendering it particularly well-suited for models like ConvMixer, where a nuanced understanding of features is critical. Therefore, the GELU function is calculated by Equation (5) as:

(5) $${ConvMixer}_{map}=GeLu\;(Concat(BNorm\left\{Convolpointwise\left(f\right)\right\},\;BNorm\left\{Convol\left(f\right)\right))$$

**Figure 4:**
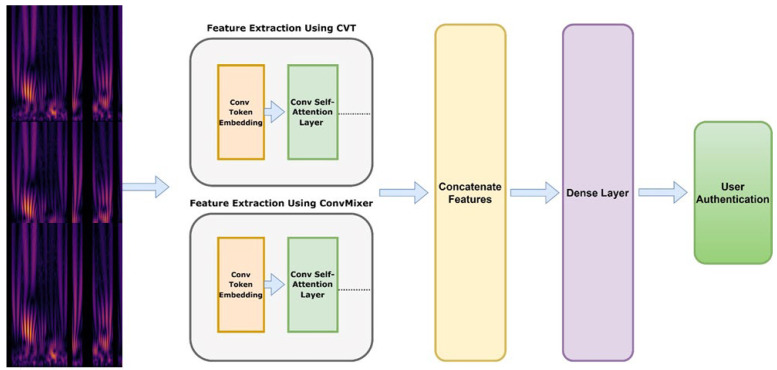
The architecture of CVT-ConvMixer model.

Features Fusion: Both CVT and ConvMixer features are fused using a concatenation or combination process. The algorithm utilizes a Flattening layer to combine these features, resulting in a comprehensive representation that benefits from both architectures. As shown in Figure 4 and describe in Algorithm 3, our attentional selective fusion (ATTSF) consists of global attention and local attention, which can add more flexibility when fusing various forms of information and is calculated by Equation (6) as:

(6) $$Dense\_Features\_map\;(i,j)={CVT}_{map}+\alpha {ConvMixer}_{map}$$

Final Classification Layer: A Dense layer is applied to the combined features to produce the final classification output. This layer employs a softmax activation function, enabling the model to output class probabilities for the input signals. Creating the Hybrid Model: The hybrid model is assembled by defining the input layer (representing the PPG signal feature vectors) and the final classification output. The entire architecture, comprising the CVT, ConvMixer, and fusion components, is encapsulated in the hybrid_model.

**Algorithm 3:** A Hybrid CVT-ConvMixer Model with Self-Attention Mechanisms
  

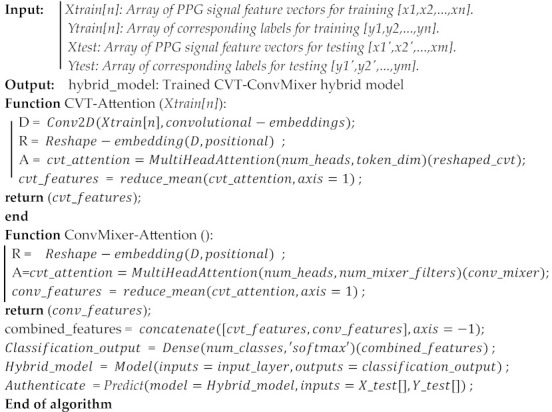



### 3.3. Model Enhancement

**Figure 5:**
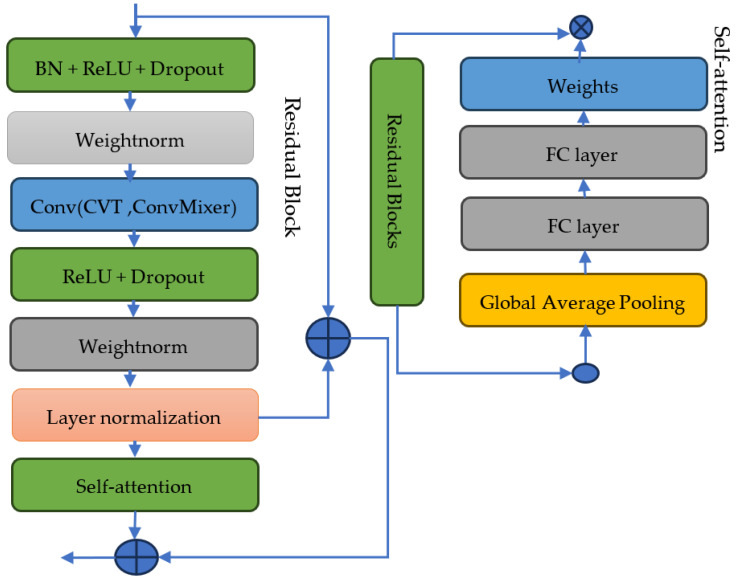
The architecture of the proposed CVT-ConvMixer model includes a residual block and a self-attention module.

Transfer Learning utilizes pre-trained models on related tasks or larger datasets to initialize the ConvMixer model’s parameters. Fine-tune the model on the PPG dataset to leverage the learned representations and potentially improve performance. Attention Mechanisms: Incorporate attention mechanisms into the ConvMixer model to allow the model to focus on the most informative parts of the PPG signal. Attention mechanisms can capture important temporal or spatial relationships, enhancing the model’s discriminative ability. By incorporating these enhancements, the accuracy, robustness, and generalization capability of the CVT-ConvMixer model for biometric authentication using PPG signals are potentially improved. Experimentation and evaluation of these techniques on specific datasets and task requirements will help determine the most effective enhancements to apply. Figure 5 shows the architecture of self-attention and residual block modules. 

To incorporate attention mechanisms into the ConvMixer model, self-attention layers capture important relationships and focus on informative parts of the PPG signals. The self-attention layers introduced in Step 6 allow the ConvMixer model to focus on informative features and capture long-range dependencies in the PPG signals. This attention mechanism helps enhance the discriminative capability of the model and can improve its performance in biometric authentication tasks. As shown in the first part of Figure 5, the focus is on the integration of self-attention layers within the ConvMixer model, specifically tailored for biometric authentication using PPG signals. The self-attention mechanism enables the model to assign varying degrees of importance to different parts of the PPG signal, facilitating a more nuanced and context-aware analysis. This is crucial for detecting intricate patterns and long-range dependencies within the data, which are essential for accurate identification and authentication. The visualization demonstrates the flow of data through these layers, highlighting the dynamic focus of the model on different aspects of the signal at various stages of processing.

The second part of Figure 5 is expected to detail the residual block modules in the ConvMixer model. Residual blocks are known for their effectiveness in combating the vanishing gradient problem and in preserving essential information throughout deep neural networks. The figure likely showcases the structure of these blocks, including the characteristic skip connections that allow the direct flow of information across layers. These connections are instrumental in maintaining the integrity of signal characteristics, ensuring that vital information is not lost during processing. By incorporating these residual blocks, the model benefits from enhanced stability and improved learning capabilities, especially for complex and deep neural architectures required in processing biometric data like PPG signals. Overall, this figure underscores the technical sophistication of the ConvMixer model, highlighting how the incorporation of self-attention and residual blocks contributes to its efficacy in biometric authentication tasks.

In our enhanced CVT-ConvMixer model, we introduce the concept of residual connections, a pivotal aspect derived from the foundational residual block architecture. These blocks are adeptly integrated into our convolutional neural network framework. Their main feature is that they can learn new features by adding things in a special way: they obtain the output o by applying a non-linear activation function π to the sum of the input *x* and the transformed output *transform (x)*, which can be written as an Equation (7).

(7) output=σ(Dense_Features_map (i,j)+transform(x))

This approach is particularly beneficial for deep networks, as it facilitates the performance of identity mapping, simplifying the learning process for complex transformations. Moreover, the CVT-ConvMixer model is further refined by embedding a sophisticated self-attention module. This module is tailored to process two distinct types of feature maps, derived from our specially constructed hybrid classifiers, including CVT and ConvMixer architectures. Because the outputs of different channels have different effects on the outcomes of predictions, we use a system to give these channels the right weights, which makes them better at making predictions. The self-attention architecture commences with a global average pooling operation on the input feature maps, producing a channel mean vector. Following a sigmoid activation, a dual-layer, fully connected network determines each channel weight *W*. The final step involves the application of these weights to the feature maps, achieved via elementwise multiplication. Equation (8) enables this by ensuring that each model’s contribution is best suited for the current prediction task.

(8) Features=W×Output

### 3.4. Biometric Identification

Biometric identification using PPG signals and the CVT-ConvMixer model is an emerging approach for identifying individuals based on their unique physiological patterns. PPG signals capture blood volume changes and can provide valuable information about cardiovascular parameters. The CVT-ConvMixer is a deep-learning architecture suitable for processing time-series data like PPG signals. The process involves collecting a dataset of PPG signals, preprocessing the data to remove noise and artifacts, and converting segmented and preprocessed signals into 2D-based images using the Scalogram technique. Afterwards, training a CVT-ConvMixer model on the dataset, extracting features from the image-based PPG signals using the trained model, and classifying the features to identify individuals. Evaluation and optimization of the system are important to improve its performance. Extensive experimentation and fine-tuning are necessary to achieve accurate and reliable biometric identification results. The following sub-sections are used to describe how to fine-tune the network to achieve higher accuracy.

### 3.5. Fine Tune Network

Fine-tuning a machine learning model involves adjusting its parameters and configurations to achieve better performance on a specific dataset. Table 2 defines the hyperparameters for the CVT-ConvMixer hybrid model for PPG-based biometric identification. The process of fine-tuning a CVT-ConvMixer hybrid model for PPG-based biometric human identification architecture is given as follows:

- Effect of Dataset Preparation: We split the dataset into three parts: training, validation, and testing sets. The training set is used to update the model’s parameters; the validation set helps in monitoring its performance during training; and the testing set evaluates the final model’s effectiveness.
- Effect of Initial Model Setup: We chose the CVT-ConvMixer hybrid architecture due to its potential to capture both spatial and temporal features in PPG signals. According to Table 2, we set hyperparameters such as segment length, embedding dimension, attention heads, and more. Next, we define the model’s input layer to accept PPG signal segments.
- Effect of Hyperparameter Tuning: We start by fine-tuning by adjusting hyperparameters like learning rate, batch size, and optimizer. The experiment with different combinations of these values is used to find the ones that result in faster convergence and improve accuracy, as described in Table 2.
- Effects of Model Architecture Modifications: Depending on the dataset’s complexity, we might consider making changes to the model architecture. We may adjust the number of layers, filters, and kernel sizes in both the CVT and ConvMixer components. These modifications aim to capture relevant patterns present in your PPG signals, as mentioned in Table 2.
- Effect of Attention Mechanisms on Fine-Tuning: The attention mechanisms play a crucial role in capturing important features. You fine-tune the parameters of the self-attention and multi-head attention layers to better extract temporal relationships and spatial dependencies within the PPG signals.
- Effects of Regularization Techniques: To avoid overfitting, we apply regularization techniques such as dropout and batch normalization. These techniques help the model generalize well to unseen data and prevent it from memorizing the training samples.
- Effect of Loss Function: We choose a suitable loss function, typically categorical cross-entropy, for your classification task. If there is a class imbalance, you may use weighted loss to give more importance to the minority class.
- Effect of Learning Rate: We implement learning rate scheduling to adjust the learning rate during training. This helps the model make finer adjustments in later epochs and prevents overshooting.
- Effect of Early Stopping and Validation: To prevent overfitting, we apply early stopping, which stops training when the validation accuracy plateaus or deteriorates. We monitor the model’s performance on the validation set to determine when to stop training.

Throughout this process, we are constantly refining the model’s settings to achieve optimal performance for PPG-based biometric-based human identification. Table 3 outlines the hyperparameters of the CVT-ConvMixer hybrid model for PPG-based biometric-based human identification. These hyperparameters are selected based on repeated experiments.

## 4. Experimental Results

### 4.1. Experimental Setup

We have collected PPG signals from three different online sources, such as the RW-PPG [31], BIDMC [32], and MIMIC [33] datasets. Initially, overlapping segments are used in authentication systems because they ensure that no crucial transitional information is lost between segments. An overlap of 50% is commonly used. For instance, if you are using 5 s segments, the next segment might start 5 s into the current one. Overlapping can help in capturing more robust features, especially if there are short-lived but significant artifacts or events in the PPG signal. Therefore, we have performed preprocessing steps to remove artifacts, as mentioned in Section 3.2.1.

The experimental work was conducted using Python and employed signal processing and deep learning libraries. The computer used for the experiments was equipped with an Intel Core i7-1884 CPU, 32 GB of RAM, and an Nvidia RTX 2080 Ti GPU. Signal parameters were determined heuristically during the initial stages of the experimentation. For the ConvMixer architecture, the chosen parameters were as follows: a patch size of 5 × 5, a hidden dimension of 32, and a mixer depth of 7. The ConvMixer was trained using the ‘sgdm’ optimizer, with a minibatch size of 64, a maximum of 7 epochs, and an initial learning rate of 0.001. The details of the hyperparameters of the CVT-ConvMixer model are defined in Table 3.

TensorFlow and Keras were utilized to develop the neural networks described in this study. The training procedure consisted of executing the networks for 100 epochs in 32, 64, and 128 batches. To prevent overfitting, training was terminated if the validation loss diverged for more than 50 epochs. During training, the mean squared error loss function was minimized. To optimize the networks, an Adam optimizer with a learning rate of 1 × 10^−3^ was utilized. Fivefold cross-validation was utilized to assess the efficacy of the models. This means that for each cycle, 20% of the data were set aside for testing, while the remaining 80% was divided into a “training + validation” set. Eighty percent of the data in the “training + validation” set was used for training, while the remaining twenty percent was used for validation.

The loss function used for the CVT-ConvMixer hybrid model for PPG-based biometric-based human identification is typically the categorical cross-entropy loss. This loss function is suitable for classification tasks where the goal is to predict one class among multiple possible classes. In your case, you have two classes: genuine and imposter. The categorical cross-entropy loss can be defined mathematically by Equation (9) as:

(9) $$Loss-Function\left(Y_{True},Y_{Pred}\right)=-\sum_{m}^{i}Y_{True}\left(i\right).log(Y_{Pred},i)$$

where, the parameter $Y_{True}$ is the true label of the sample (one-hot encoded vector), and $Y_{Pred}$ is the predicted output of the model for the sample. The CVT-ConvMixer hybrid model predicts the probability distribution of the two classes (genuine and imposter) for each PPG signal segment. The true label ($Y_{True}$) is one-hot encoded, meaning it is a binary vector where the element corresponding to the true class is 1 and the rest are 0. The predicted output ($Y_{Pred}$) is the model’s softmax output, representing the predicted probabilities for each class. This loss during the training process and use it to update the model’s parameters using gradient descent or any suitable optimization algorithm.

### 4.2. Statistical Analysis

It is worth noting that these metrics are typically used with other evaluation measures, such as accuracy, precision, and ROC curves, to comprehensively analyze the model’s performance. Sensitivity, specificity, and F1-score are commonly used statistical metrics for evaluating the performance of classification models. These metrics provide insights into the model’s ability to correctly identify positive and negative instances. In biometric authentication using PPG signals, a positive instance is the correct identification of an individual, while a negative instance is the accurate rejection of an unmatching or unknown biometric signal, affirming the system’s precision and reliability. Here is a description of each metric:

1. Sensitivity (also known as Recall or True Positive Rate): indicates the model’s ability to detect positive instances and is useful when the identification of true positives is important. It measures the proportion of actual positive instances correctly identified by the model. It is calculated as the ratio of true positives (*TP*) to the sum of true positives and false negatives (*FN*), as in the following Equation (10):
  (10) $$Sensitivity=\frac{TP}{TP+FN}$$
2. Specificity: indicates the model’s ability to correctly identify negative instances and is valuable when correctly identifying true negatives is critical. It measures the proportion of actual negative instances correctly identified by the model. It is calculated as the ratio of true negatives (*TN*) to the sum of true negatives and false positives (*FP*), as in the following Equation (11):
  (11) $$Specifity=\frac{TN}{TN+FP}$$
3. F1-score: The F1-score considers both the model’s ability to correctly identify positive instances (*recall*) and the proportion of correctly identified positive instances out of all instances labeled as positive (*precision*). The F1-score provides a single metric that balances the trade-off between precision and recall, making it useful in scenarios where both metrics are important. The F1-score is a combined metric that balances both precision and recall (sensitivity). It is calculated as the harmonic mean of precision and recall as in the following Equation (12):
  (12) $$F1-score=2\times \frac{\left(Precision\times Recall\right)}{Precision+Recall}$$

These metrics are particularly relevant in binary classification tasks, where there are two classes: positive and negative. Sensitivity and specificity focus on the performance of the model in each class separately, while the F1-score provides an overall evaluation by considering both classes simultaneously. In addition, we have used the area under the receiver operating curve (AUC) to show the performance of the proposed system.

### 4.3. Cross-Validation on RW-PPG, BIDMC, and MIMIC

To cross-validate using the three datasets, RW-PPG [31], BIDMC [32], and MIMIC [33], we trained on one dataset and tested on the other two, rotating through each dataset. First, take the RW-PPG dataset: Use it for training and testing models on both the BIDMC and MIMIC datasets. This gives us an idea of how well the model, trained on RW-PPG, performs on the other two datasets. Next, move to the BIDMC dataset: Train the model and then evaluate its performance on the RW-PPG and MIMIC datasets. This shift allows us to see how training on BIDMC influences the model’s ability to recognize patterns in the other datasets. Finally, use the MIMIC dataset for training. Once the model is trained, test it on the RW-PPG and BIDMC datasets. This last round completes the cycle, ensuring each dataset has served as a training set once.

After we have run all three rounds, review the performance metrics. These results tell us how adaptable and versatile the proposed model is across different data sources. Using this cross-validation method ensures a more robust model, as it is been validated across diverse datasets, reducing potential biases and overfitting.

**Figure 6:**
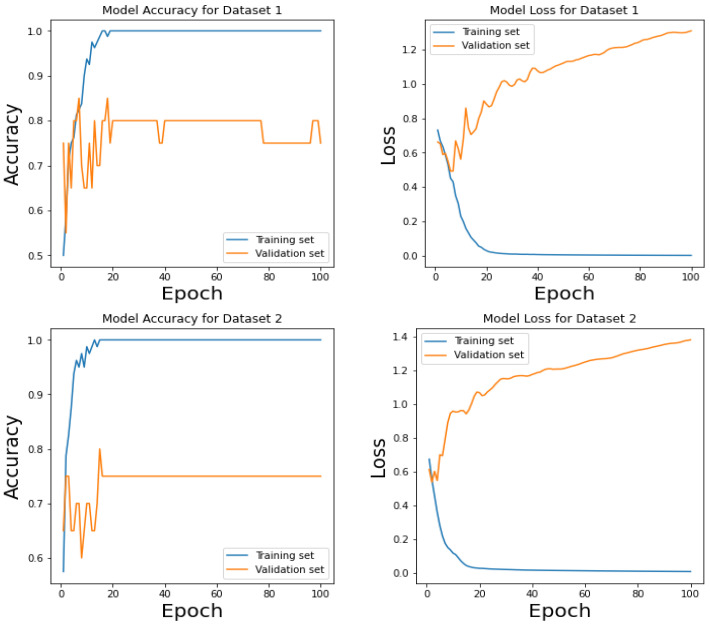
Training and validation versus loss with respect to epoch curves without a fine-tuned network on datasets 1 and 2. where dataset 1 refers to RW-PPG [31] and Beth Israel Deaconess Medical Center (BIDMC) [32], and dataset 2 refers to Multiparameter Intelligent Monitoring for Intensive Care (MIMIC) [33].

**Figure 7:**
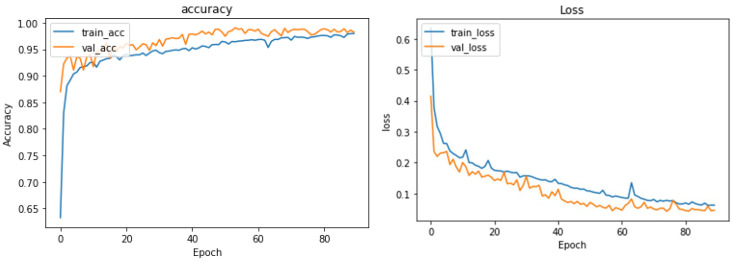
Curves for training and validation versus loss with respect to epochs with parameters fine-tuned on a combination of RW-PPG [31], BIDMC [32], and MIMIC [33] PPG datasets.

**Figure 8:**
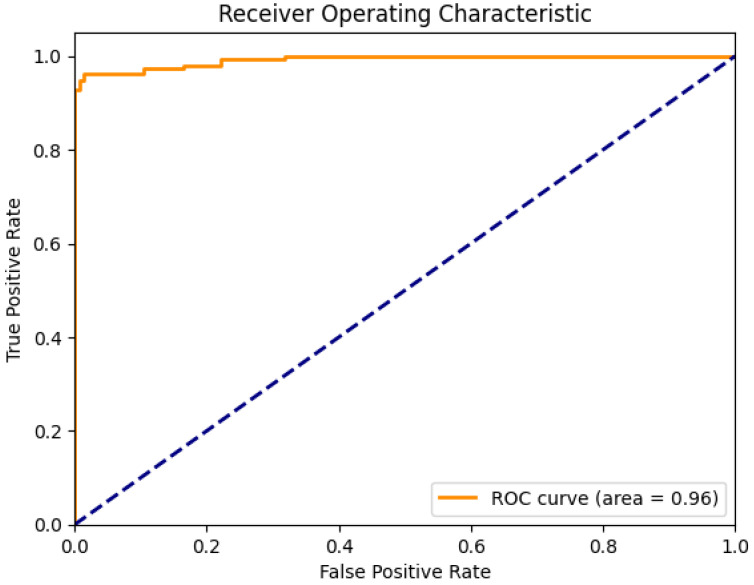
AUC curve for human identification based on collected PPG signals from three datasets such as RW-PPG [31], BIDMC [32], and MIMIC [33].

Figure 6 shows the training and validation versus loss as to epochs. The AUC curve in Figure 7 demonstrates that the proposed system, with the preprocessing step, outperforms human identification based on PPG signals. In this paper, we have fixed 80 epochs of training and validation versus loss as epochs, as shown in Figure 8.

### 4.4. Results Exploration

To evaluate the performance of the proposed system, we have used four different hyper-parameter settings, as mentioned in Table 4.

Table 5 presents the experimental results for Setting 1 of the CVT-ConvMixer model, evaluating its performance on various metrics. In this table, we assess the model’s accuracy, sensitivity, specificity, F1-score, and loss across three different datasets: training, validation, and testing. The accuracy values of 0.95 suggest that the model correctly predicted 95% of the instances in all three datasets. Similarly, high sensitivity and specificity scores of 0.92 and 0.97, respectively, indicate that the model effectively identified positive and negative cases. The F1-score of 0.93 demonstrates a good balance between precision and recall. Lastly, the low loss value of 0.15 indicates that the model’s predictions are close to the actual values, reflecting its overall robust performance across the different datasets.

**Figure 9:**
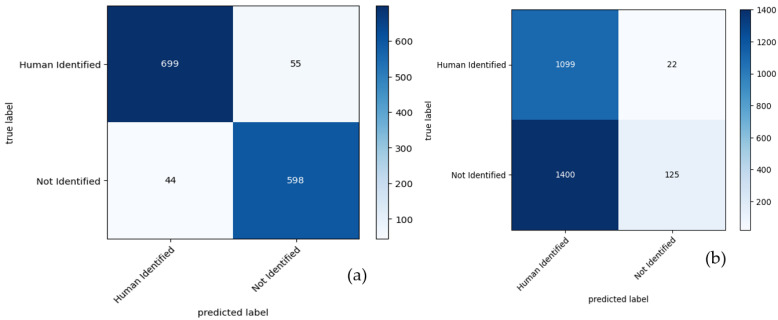
Confusion matrix for results obtained by a proposed CVT-ConvMixer model for biometric based human identification, where figure (**a**) shows results without a fine-tune network and figure (**b**) represents a fine-tune network on three datasets such as RW-PPG [31], BIDMC [32], and MIMIC [33].

Figure 9 consists of two parts, (a) and (b), which display confusion matrices for the results obtained by a proposed CVT-ConvMixer model used in biometric-based human identification. These confusion matrices are typically used to assess the performance of a classification model, particularly in scenarios where you want to understand how well the model is distinguishing between different classes (in this case, individuals). Figure 9 provides a visual representation of the model’s performance in recognizing individuals in a biometric-based human identification system. It helps to evaluate how well the model distinguishes between different individuals and assesses the impact of fine-tuning on its accuracy.

Table 6 and Table 7 provide the experimental results for Setting 2, Setting 3, and Setting 4 of the CVT-ConvMixer model. Table 5, Table 6 and Table 7 present the experimental results for different settings of the CVT-ConvMixer model, each evaluated on various performance metrics. In Table 5, for Setting 2, the model exhibits high accuracy (0.96) on the training data, demonstrating its ability to correctly classify instances. Additionally, it shows a strong balance between sensitivity (0.93) and specificity (0.98), highlighting its effectiveness in identifying both positive and negative cases. The F1-score (0.94) indicates good overall model performance, while the low loss (0.10) suggests that the model’s predictions closely align with the actual values. Similar patterns are observed for Table 6 and Table 7, representing Settings 3 and 4, with slightly varying hyperparameter performance but consistently strong results across training, validation, and test datasets. These tables collectively showcase the robustness and reliability of the CVT-ConvMixer model across different configurations and evaluation sets.

Table 8 presents experimental results for Setting 4 of the CVT-ConvMixer model, showcasing its performance on the dataset. The model’s overall classification accuracy is high across all datasets, with 95% accuracy on training data, 94% on validation data, and 96% on the test data. This indicates that the model correctly classified the majority of instances. The model exhibits strong sensitivity, or recall, with values of 0.93 for training, 0.94 for validation, and 0.93 for the test dataset. This implies the model’s ability to correctly identify positive instances, which is especially important for tasks where identifying true positives is critical. The model demonstrates high specificity, achieving values of 0.97 for training, 0.96 for validation, and 0.95 for the test dataset. High specificity means the model effectively identifies negative instances. The F1-score, which balances precision and recall, indicates that the model performs well in finding the right balance between minimizing false positives and false negatives. It reaches an F1-score of 0.96 for training, 0.94 for validation, and 0.95 for the test dataset. The loss values are low, measuring 0.02 for training, 0.11 for validation, and 0.12 for the test dataset. Low loss values suggest that the model’s predictions align well with the actual data. Hence, the CVT-ConvMixer model in Setting 4 demonstrates strong overall performance across various evaluation metrics, indicating its effectiveness in classification tasks. It achieves high accuracy, sensitivity, and specificity while maintaining a balanced F1-score and low loss values on both training and validation datasets. These results suggest that the model is well-suited for its intended task.

**Figure 10:**
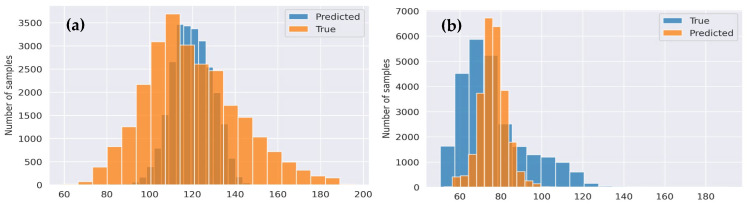
The comparison results of the proposed (**a**) CVT-ConvMixer model for biometric-based human identification shows outperform compared to and (**b**) basic ConvMixer model in terms of predicted and true datasets on three datasets, such as RW-PPG [31], BIDMC [32], and MIMIC [33].

Figure 10 presents a comparison of two models in the context of biometric-based human identification. Figure 10a shows that the proposed CVT-ConvMixer model achieves better results compared to another model (ConvMixer). The model compares predicted values (model’s classifications) against true values (ground truth) in terms of performance metrics. We employed metrics such as the mean squared error (MSE). This metric provides a quantifiable measure of the difference between the two distributions. In fact, the MSE gives an average of the squares of the errors, indicating the variance between predicted and true values. This graph indicates that the CVT-ConvMixer model excels in the biometric identification task.

**Figure 11:**
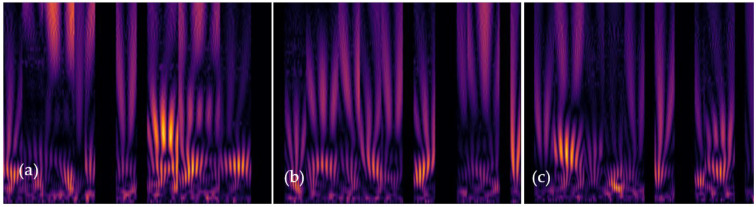
Pattern Patches extracted by Scalogram are identified by the proposed (CVT-ConvMixer) model through four individual samples of each person based on 5 s of PPG signals, where figure (**a**) shows person 1, figure (**b**) represents person 2, and figure (**c**) shows person 3 from the RW-PPG [31] dataset.

Figure 11 illustrates the pattern patches extracted by a scalogram and identified by the proposed CVT-ConvMixer model for different individuals using samples of PPG (Photoplethysmogram) signals of duration (5 s). This figure provides insights into how the model distinguishes individuals based on the patterns it extracts from these signals. The description mentions three individuals (Person 1, Person 2, and Person 3) represented in the figures. This model stands out for its speed and efficiency, key attributes for real-time applications where low latency is crucial. To prove the low latency, we have described it in Section 4.5. Unlike more intrusive biometric methods, the use of PPG signals allows for a non-intrusive and continuous form of user verification, ideal for scenarios like secure access control or ongoing patient monitoring in healthcare settings. The model’s proficiency in capturing unique physiological patterns inherent in cardiovascular signals suggests robustness and adaptability to variations in human physiological states, an essential feature for dynamic environments. This is achieved by converting PPG signals into 2-D scalograms and extracting their robust features. 

In Figure 11, the patterns extracted by the scalogram and identified by the CVT-ConvMixer model are likely distinctive features or characteristics within the PPG signals that enable the model to differentiate between individuals. These patterns might be related to the unique physiological aspects of each person’s cardiovascular system, which can be captured by analyzing PPG data. Accordingly, Figure 11 visually showcases the patterns extracted from PPG signals and how the proposed CVT-ConvMixer model uses these patterns to identify different individuals, offering insights into the model’s capabilities for biometric-based person identification.

**Figure 12:**
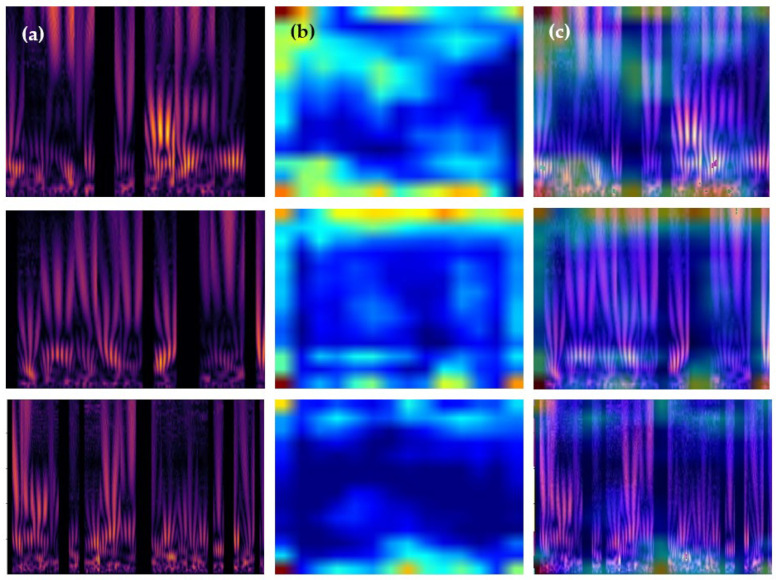
A Grad-CAM analysis of the proposed (CVT-ConvMixer) model through four individual samples of each person based on 5 s of PPG signals, where figure (**a**) shows the original input of a 2D-scalogram of person 1, person 2, person 3, figure (**b**) represents heatmaps, and figure (**c**) shows the corresponding superimposed images from the RW-PPG [31] dataset.

Figure 12 in our study illustrates the Gradient-weighted Class Activation Mapping (Grad-CAM) analysis of our CVT-ConvMixer model, applied to PPG 2D-scalogram signals. Figure 12a shows the original 2D-scalograms for three individuals, highlighting the model’s input; (b) displays heatmaps generated by Grad-CAM, pinpointing regions crucial for the model’s predictions; and (c) presents superimposed images, merging the original scalograms with their heatmaps to visualize the model’s focus areas. This figure demonstrates the model’s proficiency in identifying and differentiating key biometric patterns within the PPG signals, underscoring its effectiveness in biometric authentication.

### 4.5. Comparisons with Literature

There were state-of-the-art techniques that used PPG signals to identify humans. We have compared these systems, such as CNN-RNN [11], CNN [20], CNN-LSTM [21], and Fuzzy-Min-Max NN [23], to the proposed system. Those comparisons are described in the following paragraphs. To perform comparisons, we have implemented these systems, and we used our preprocessing steps to enhance PPG signals.

Table 9 compares state-of-the-art biometric identification systems with the CVT-ConvMixer model on three datasets, such as RW-PPG [31], BIDMC [32], and MIMIC [33]. The first model, denoted as [11], is a combination of Convolutional Neural Networks (CNN) and Recurrent Neural Networks (RNN), referred to as CNN-RNN. This model achieved an accuracy of 0.90, exhibiting a sensitivity of 0.93 and a specificity of 0.92. Furthermore, its F1-score and AUC were recorded as 0.91 and 0.90, respectively. Following the CNN-RNN is the CNN model from the study [20]. By solely utilizing the CNN approach, this model managed an accuracy of 0.85. Its sensitivity and specificity were determined to be 0.86 and 0.85, respectively, while its F1-score stood at 0.83 and the AUC at 0.85. A study [21] introduced a fusion of CNN with Long Short-Term Memory (LSTM) networks, aptly named CNN-LSTM. This model outperformed the previous two with an impressive accuracy of 0.96. Though its sensitivity is 0.93, it boasts a specificity of 0.95. The F1-score mirrors its accuracy at 0.95, but the AUC lags slightly at 0.93.

The model labeled [23], Fuzzy-Min-Max NN, deploys a different approach using a Fuzzy-Min-Max Neural Network. However, in comparison to the models, it reflects lower performance. Its accuracy is 0.78, with sensitivity and specificity recorded as 0.80 and 0.82, respectively. Both the F1-score and AUC for this model are noted to be 0.81. Lastly, the CVT-ConvMixer model is another significant contender on the list. It exhibits accuracy nearly on par with the CNN-LSTM at 0.95. What is notable is its outstanding sensitivity of 0.97, coupled with a specificity of 0.95. The F1-score for this model is 0.95, and its AUC is slightly higher than its accuracy, at 0.96.

In conclusion, this table presents a detailed yet concise comparison, shedding light on the capabilities and efficiencies of various biometric identification models when tested on the three datasets: RW-PPG, BIDMC, and MIMIC. Among them, the CNN-LSTM and CVT-ConvMixer models stand out as particularly potent, while the Fuzzy-Min-Max NN lags in performance.

In Table 10, we are presented with a comparative view of the computational performance of various biometric identification models, namely CNN-RNN [11], CNN [20], CNN-LSTM [21], Fuzzy-Min-Max-NN [23], and CVT-ConvMixer, against three data sets such as RW-PPG [31], BIDMC [32], and MIMIC [33]. The comparison is depicted across three different hardware configurations, including CPUs, GPUs, and TPUs, offering a lens through which the training and testing times of these models can be evaluated.

The CNN-RNN [11] model demonstrates a range of training times, from 1050 s when utilizing a TPU to 1300 s on a CPU. In contrast, the testing times are slightly more condensed, presenting a spread from 110 s on a TPU to 130 s when evaluated on a CPU. Following this, the CNN [20] model exhibits an appreciable swiftness in computation, with training durations spanning from 950 s on TPU to 1200 s on CPU, while the testing phases are relatively concise, spanning 80 s to 99 s on the same respective hardware configurations. Interestingly, the CNN-LSTM [21] model manifests training times that, while relatively proximate to the CNN-RNN model, show minute enhancements in efficiency across certain benchmarks. With training times bracketed between 1100 s on TPU and 1328 s on CPU and testing times delineated from 100 s to 120 s on the same respective platforms, subtle yet discernible distinctions in computational efficacy are observed.

On the other hand, the Fuzzy-Min-Max-NN [23] model illustrates a peculiar computational profile in which it bears the most prolonged training times among its peers, ranging from 1300 s on TPU to a peak of 1500 s on CPU. Conversely, its testing times are rendered to be relatively lower, between 70 s on TPU and 90 s on CPU, which might indicate an inverse relationship between its training and testing computational efficiencies. In stark contrast, the CVT-ConvMixer model emerges conspicuously as the most computationally efficient or low latency among the array of models, evidencing training times that oscillate from a mere 350 s on TPU to 700 s on CPU and testing times that are even more notably succinct, stretching from 35 s on TPU to 70 s on CPU. This starkly contrasts the computational demands of its counterparts and positions it as a notably efficient model in both the training and testing phases of operation.

In light of these observations, we can deduce that the CVT-ConvMixer model not only substantiates itself as significantly more computationally efficient across both training and testing paradigms when juxtaposed with the other models but also that, uniformly, TPUs appear to offer the most time-efficient hardware across both operational phases for all models. This invaluable data underscores the pivotal role that models and hardware selection can play in optimizing biometric identification tasks and illuminates pathways for future research and practical applications in the field.

## 5. Discussions

With the growing prevalence of the Internet of Health Things [25], ensuring robust security policies to safeguard sensitive data has become crucial. Traditional methods of user authentication may not be sufficient to protect against unauthorized access during data collection, storage, and transmission. Therefore, there is a need for advanced technologies that leverage unique personal identifiers, such as biometric characteristics, for user authentication. This paper introduces a robust PPG-based authentication system capable of authenticating users as opposed to other authentication systems due to the use of preprocessing, scalogram, and feature extraction techniques using a hybrid classifier. The proposed system consists of several stages, including preprocessing and filtering, motion artifact removal, template and feature extraction, and training. To enhance the reliability of the extracted features, a multiwavelet-based feature extraction mechanism is employed, which outperforms conventional scalar-wavelet schemes and allows the learning model to better differentiate between users. Additionally, an autoencoder is utilized to transform the input PPG signal into a latent space, further improving the authentication process. Various distance measures can be employed for user classification or authentication. To create a comprehensive dataset, three public datasets are combined with a locally collected dataset, resulting in a total of 120 subjects.

Recent studies have shown promising results in using photoplethysmography (PPG) [30] signals for biometric recognition to enhance the security of mobile devices. However, these methods typically process PPG signals as one-dimensional data. In other application scenarios, feature extraction techniques based on transformations of the spectrogram have been successful in improving signal processing accuracy. This paper presents a preliminary study on a biometric recognition approach that extracts features from different transformations of the 2D-Scalogram of PPG signals and utilizes machine learning techniques for classification. Our PPG-based biometric authentication system effectively distinguishes genuine users from imposters using unique biometric signatures derived from PPG signals, enhanced by advanced preprocessing and multiwavelet-based feature extraction. The transformation into 2-D scalograms allows detailed signal analysis, while the hybrid CVT-ConvMixer classifier with attention mechanisms focuses on relevant features, enhancing accuracy. High scores in sensitivity, specificity, F1-score, and AUC demonstrate its proficiency. A comprehensive dataset ensures robust performance across diverse populations, making our system reliable for real-world applications in user verification.

To the best of our knowledge, this is the first study in the literature that extracts features from the spectrogram of PPG signals for biometric systems. In addition, unlike many state-of-the-art biometric recognition techniques, the proposed approach does not require the search for fiducial points, thereby reducing computational complexity and increasing the robustness of the signal preprocessing step.

Biometric authentication stands as a widely adopted means of verifying individuals’ identities. The integration of Photoplethysmography (PPG) with deep learning techniques holds immense promise for augmenting its efficacy. PPG, a non-invasive optical method measuring blood volume changes, synergizes with deep learning—utilizing artificial neural networks for pattern recognition. Our endeavor involves crafting a biometric authentication system employing PPG signals. Leveraging feature fusion from the CVT-ConvMixer hybrid classifier, we achieved numerous benefits, such as robustness of feature extraction and performance improvements.

Our methodology encompasses several stages. The journey begins with the preprocessing of PPG data, paving the way for feature extraction using the CVT-ConvMixer model. The infusion of attention mechanisms further refines the process, honing the classification of human identities. Through rigorous experimentation and the evaluation of a comprehensive PPG dataset, our approach’s efficacy shines. We harness metrics such as sensitivity, specificity, and F1-score to meticulously gauge the model’s competence in precisely discerning legitimate individuals.

An especially noteworthy facet is the integration of attention mechanisms within the ConvMixer model. This strategic inclusion empowers the model with heightened discriminative capabilities. The model adeptly captures pivotal temporal relationships and informative attributes embedded within the intricate PPG signals.

Empirical validation is a cornerstone of our work. Through exhaustive experiments across three distinct PPG datasets, our proposed method garners remarkable recognition rates: an impressive accuracy (ACC) of 98.65%, a commendable sensitivity (SE) of 97.76%, and a robust specificity (SP) of 99.69%. These outcomes demonstrate that our proposed system outperformed in the realm of PPG signal processing. The recognition rates underscore the methodology’s effectiveness in proficiently classifying and identifying patterns latent within PPG signals.

The approach’s assets—data availability; simplicity; and robustness—render it a compelling fit for domains like smart healthcare and telehealth. The potential to revolutionize authentication in real-world settings imbues this approach with great promise. Comparing the CVT-ConvMixer model with state-of-the-art techniques in biometric authentication using PPG signals requires considering the latest advancements in the field. While we cannot provide real-time information, we can highlight some common state-of-the-art techniques and factors to consider in the comparison:

- State-of-the-art techniques often involve advanced deep learning architectures tailored for biometric authentication. These may include hybrid models combining multiple types of neural networks, such as Convolutional Neural Networks (CNNs), Recurrent Neural Networks (RNNs), Transformers, or Capsule Networks. These architectures aim to capture complex patterns and dependencies in PPG signals, leveraging the strengths of different models.
- Attention mechanisms have gained popularity in biometric authentication systems as they allow the model to focus on the most relevant parts of the PPG signal. State-of-the-art techniques incorporate attention mechanisms to enhance feature extraction and improve the discriminative power of the model.
- PPG signals can vary across different individuals, populations, or devices. State-of-the-art techniques may include domain adaptation methods to address the challenge of domain shift and improve the model’s generalization across different datasets or settings.
- State-of-the-art techniques often leverage large-scale PPG datasets for training and evaluation. These datasets enable models to learn complex representations and improve performance in real-world scenarios.

When comparing the CVT-ConvMixer model with state-of-the-art techniques, consider these factors along with specific performance metrics, such as accuracy, sensitivity, specificity, F1-score, and computational efficiency. It is important to refer to recent research papers and scientific literature to stay up-to-date with the latest advancements in biometric authentication using PPG signals and identify the most competitive techniques in the field, as described in Table 9. The advantages and disadvantages listed in Table 11 vary depending on specific implementation details and datasets. It is important to evaluate and compare the models based on specific requirements and the characteristics of the biometric authentication task using PPG signals.

PPG signals, reflecting blood volume changes, are indeed complex and can be influenced by various physiological conditions like stress, health status, etc. A thorough analysis considering these dynamic behaviors under various physiological contexts would typically be crucial to validating and understanding the model’s applicability and reliability across diverse real-world scenarios. in the context of requiring several seconds of PPG signal to discern differences between individuals raises valid considerations. Continuous authentication typically refers to perpetually verifying the user’s identity during the duration of a session or interaction, ensuring that the user remains an authenticated individual. The practicality and efficacy of such an approach would indeed depend on the model’s ability to discern individual differences rapidly and reliably from the PPG signal in real-time or near-real-time scenarios. Addressing these points in future work might involve

When implementing biometric authentication using PPG signals and the CVT-ConvMixer model, several challenges may arise. These challenges can impact the performance, reliability, and generalizability of the system. Here are some common challenges to consider:

- PPG signal quality can vary due to factors such as sensor placement, motion artifacts, noise interference, and physiological variations. Ensuring high-quality data acquisition is crucial for accurate and reliable authentication.
- PPG signals can be affected by environmental conditions, such as changes in lighting, temperature, or ambient noise. Adapting the system to different environments and accounting for such variations is necessary for robust authentication.
- PPG signals may exhibit inter-individual differences due to factors like age, health conditions, and anatomical variations. The system should accommodate individual variability to avoid biased or discriminatory authentication.
- The system’s performance should be evaluated across diverse populations to ensure its effectiveness across different demographics. Generalizing the authentication capabilities to a broader range of individuals is a challenge that requires careful consideration.

Addressing these challenges requires a multidisciplinary approach that encompasses signal processing, machine learning, data management, and user-centric design principles. Ongoing research and development efforts are essential to overcome these challenges and advance the field of biometric authentication using PPG and the CVT-ConvMixer model.

## 6. Conclusions and Future Directions

Our study establishes a powerful synergy between Photoplethysmography (PPG) and deep learning techniques for advancing biometric authentication. By fusing the capabilities of the Convolutional Vision Transformer (CVT) and ConvMixer architectures, we have engineered a novel hybrid classifier. This innovative approach capitalizes on the strengths of both architectures, resulting in a model with enhanced representational power, synergistic information extraction, and robustness. Through a meticulously structured methodology, we demonstrated the effectiveness of our approach in preprocessing PPG data, extracting meaningful features, and leveraging attention mechanisms for classification. Rigorous experimentation with diverse PPG datasets validated the proposed system. Metrics such as accuracy, sensitivity, and specificity showcased remarkable recognition rates, highlighting the model’s proficiency in accurately distinguishing individuals. An integral highlight of our work lies in the integration of attention mechanisms within the ConvMixer model. This strategic augmentation not only enhances discriminative capabilities but also captures crucial temporal relationships and informative nuances embedded in PPG signals. The impressive recognition rates achieved underscore the potential impact of our approach. Its applicability extends beyond the confines of our study, offering simplicity, robustness, and data availability that align with smart healthcare and telehealth paradigms. In essence, our study bridges the domains of biometric authentication, PPG signal processing, and deep learning. The synthesized approach stands as a promising avenue for elevating the accuracy, reliability, and practicality of identity verification in real-world scenarios.

The potential future directions can build upon the challenges and limitations of this study, contributing to the advancement of PPG-based biometric authentication and deep learning techniques. The future directions are listed below.

- Investigate advanced attention mechanisms that can capture more intricate temporal relationships and spatial dependencies within PPG signals. This could lead to even more precise feature extraction and improved classification accuracy.
- Explore the integration of PPG signals with other biometric modalities, such as ECG or accelerometer data. This could result in a more comprehensive and robust authentication system by leveraging multiple physiological sources of information.
- PPG signals, reflecting blood volume changes, are indeed complex and can be influenced by various physiological conditions like stress, health status, etc.
- Investigate methods to make the model more resilient to noisy or incomplete PPG signals. Techniques such as data augmentation, robust training strategies, and denoising algorithms could enhance the model’s performance in challenging conditions.

## Figures and Tables

**Table 1:** Strengths and limitations of latest relative studies.

Study	Learning Model	Dataset	Accuracy	Strengths	Limitations
[11]	CNN-RNN	TROIKA	96%	Captures sequential information	Small dataset size, not compared to other DL models
[12]	Ensemble Siamese Network (ESN)	ECG-ID and PTB	93.6%	Authentication system with high accuracy and robustness	Small dataset size, not compared to other DL models
[13]	Incorporating DL with various CNN architectures	PPG-DaLiA	93.2%	Handle motion artifact compensation and scenario-specific optimizations	May be susceptible to environmental variations and occlusions
[14]	A CNN model is presented in the study to identify unique and time-stable features	PRRB, TROIKA, Biosec1 and Biosec2	-	Effective authentication	Limited focus on biometric identification security
[16]	Homomorphic RF	Five datasets	93.6%	Protection of biometric privacy	Higher computational overhead due to homomorphic encryption
[17]	Siamese Network	Private	94%	Multicycle averaging preserves individuality	Optimal number of cycles may vary depending on individuals
[19]	Dual-Domain and Multiscale Fusion DNN	Private	98.7%	Enhances accuracy and security	Performance on datasets with diverse characteristics was not evaluated
[20]	CNN	Troika and PulseID	83.2%	Utilizes physiological factors for feature selection	Limited focus on biometric identification security
[21]	CNN-LSTM		87.1%	Effective continuous authentication	Long inference times due to high model complexity
[23]	Fuzzy Min-Max NN	Capnobase	97.62%	Utilizes liveness detection	Evaluation limited to the Capnobase database
[24]	Combined CNNs with FL.	Private	90%	Ensures data privacy and management	Resource-intensive and complex setup for federated learning
[25]	Autoencoders and LOF	CapnoBase and BIMDC datasets	91.3%	High performance with a simple approach	Limited evaluation of other benchmark datasets
[26]	Bi-LSTM	Private	94%	Feature-based and raw data-based approaches	Comparison with other state-of-the-art approaches is not provided

**Table 2:** A table summarizing the three datasets mentioned (RW-PPG, BIDMC, and MIMIC) provides information about the number of recordings, sampling frequency, and signal durations in each dataset.

Dataset Name	Number of Recordings	Observations in Each Record	Recorded Signal Durations	Sampling Frequency
RW-PPG [31]	35	50	5 s	225 Hz
BIDMC-PPG-Signals [32]	53	200	8 min	125 Hz
MIMIC-PPG-Signals [33]	32	100	5 min	125 Hz

**Table 3:** Hyperparameters are defined for the CVT-ConvMixer hybrid model for PPG-based biometric identification.

Hyperparameter	Description	Value
Segment Length	Length of each PPG signal segment	128
Number of Channels	Number of channels in the PPG signal	1
Embedding Dimension	Dimension of the embedded features	64
Convolution Kernel Size	Size of the convolutional kernel	3
Number of Tokens	Number of tokens in the reshaped CVT output	16
Token Dimension	Dimension of each token in the reshaped CVT output	4
Number of Attention Heads	Number of attention heads in MultiHeadAttention	4
Number of Mixer Filters	Number of filters in ConvMixer	32
Mixer Kernel Size	Size of the ConvMixer kernel	5
Number of Classes	Number of classes for classification	2
Number of Epochs	Number of training epochs	10
Batch Size	Number of samples in each training batch	32
Learning Rate	Initial learning rate for optimization	0.001
Dropout Rate	Probability of dropout during training	0.2
L2 Regularization	Strength of L2 regularization	0.01
Learning Rate Scheduler	Type of learning rate schedule	Exponential
Early Stopping	Whether to use early stopping	True

**Table 4:** Hyperparameter settings to test the performance of the CVT-ConvMixer model.

Hyperparameter	Setting 1 *	Setting 2 *	Setting 3 *	Setting 4 *
Number of Convolutional Layers	3	4	5	3
Kernel Size	3	5	7	5
Number of Filters	32	64	128	64
Dropout Rate	0.2	0.4	0.5	0.3
Pooling Operation	MaxPooling	MaxPooling	AvgPooling	MaxPooling
Activation Function	ReLU	ReLU	LeakyReLU	ReLU
Learning Rate	0.001	0.01	0.001	0.005
Batch Size	32	64	128	64
Number of Epochs	50	100	75	80
Weight Initialization	Random	Xavier	He	He

* Setting 1, 2, 3, and 4 are experimental settings to evaluate the performance of the proposed architecture.

**Table 5:** Average experimental results for Setting 1 of the CVT-ConvMixer model on three datasets such as RW-PPG [31], BIDMC [32], and MIMIC [33].

Hyperparameter	Accuracy	Sensitivity	Specificity	F1-Score	Loss
Training	0.95	0.92	0.97	0.93	0.15
Validation	0.95	0.92	0.97	0.93	0.15
Test	0.95	0.92	0.97	0.93	0.15

**Table 6:** Average experimental results for Setting 2 of the CVT-ConvMixer model on three datasets, such as RW-PPG [31], BIDMC [32], and MIMIC [33].

Hyperparameter	Accuracy	Sensitivity	Specificity	F1-Score	Loss
Training	0.96	0.93	0.98	0.94	0.10
Validation	0.95	0.92	0.97	0.93	0.11
Test	0.95	0.92	0.97	0.93	0.13

**Table 7:** Average experimental results for Setting 3 of the CVT-ConvMixer model on three datasets such as RW-PPG [31], BIDMC [32], and MIMIC [33].

Hyperparameter	Accuracy	Sensitivity	Specificity	F1-Score	Loss
Training	0.94	0.92	0.96	0.95	0.05
Validation	0.95	0.92	0.97	0.93	0.15
Test	0.95	0.92	0.97	0.93	0.15

**Table 8:** Experimental results for Setting 4 of the CVT-ConvMixer model.

Hyperparameter	Accuracy	Sensitivity	Specificity	F1-Score	Loss
Training	0.95	0.93	0.97	0.96	0.02
Validation	0.94	0.94	0.96	0.94	0.11
Test	0.96	0.93	0.95	0.95	0.12

**Table 9:** State-of-the-art biometric identification system compared to the CVT-ConvMixer model on three datasets, such as RW-PPG [31], BIDMC [32], and MIMIC [33].

Studies	Accuracy	Sensitivity	Specificity	F1-Score	AUC
CNN-RNN [11]	0.90	0.93	0.92	0.91	0.90
CNN [20]	0.85	0.86	0.85	0.83	0.85
CNN-LSTM [21]	0.96	0.93	0.95	0.95	0.93
Fuzzy-Min-Max-NN [23]	0.78	0.80	0.82	0.81	0.81
CVT-ConvMixer	0.95	0.97	0.95	0.95	0.96

**Table 10:** State-of-the-art biometric identification system in terms of computational time compared to the CVT-ConvMixer model on three datasets, such as RW-PPG [31], BIDMC [32], and MIMIC [33] based on different benchmarks.

Studies	Training(s) CPU/GPU/TPU	Testing(s) CPU/GPU/TPU
CNN-RNN [11]	1300/1100/1050	130/120/110
CNN [20]	1200/1000/950	99/89/80
CNN-LSTM [21]	1328/1123/1100	120/110/100
Fuzzy-Min-Max-NN [23]	1500/1350/1300	90/80/70
CVT-ConvMixer	700/500/350	70/50/35

**Table 11:** Compare Transfer Learning with the CVT-ConvMixer model to other models in the context of biometric authentication using PPG signals.

Model	Advantages	Disadvantages
CVT and ConvMixer	Captures temporal dependencies effectively. Efficient parallel processing. Can incorporate attention mechanisms.	Training time and quality of PPG signals.
Transfer Learning	Enables knowledge transfer from related tasks. Helps with data efficiency. Improves generalization capabilities. Saves computation time and resources	It requires a suitable pre-trained model; a pre-trained model may not perfectly align. The fine-tuning process may be time-consuming. Performance heavily depends on source task.
CNN	Strong performance in image-based tasks. Well-established architectures and resources Interpretable feature hierarchies	Limited ability to capture temporal dynamics. May require larger computational resources. May be susceptible to overfitting.
LSTM	Captures long-term dependencies in sequences. Suitable for modeling temporal dynamics. Handles variable-length sequences.	Computationally more demanding. Prone to vanishing/exploding gradient problem. Limited parallel processing capability.
Capsule Networks	Captures hierarchical relationships between entities. Robust to viewpoint variations. Potentially better handling of complex patterns.	Limited availability of pre-trained models. Higher computational complexity. Requires larger amounts of labeled data.
Siamese Networks	Effective for one-shot or few-shot learning. Handles limited training data. Can verify identities with a small number of samples.	Limited by the availability of labeled pairs. Performance heavily relies on the similarity metric. May require complex architecture.

## Data Availability

To help the research community, the code utilized in this paper for the CVT-ConvMixer model with preprocessing of PPG signals is freely available online at Github (https://github.com/Qaisar256/Biometric-PPG) (accessed on 4 December 2023). Whereas the PPG datasets are available at: RW-PPG [33]: https://data.mendeley.com/datasets/yynb8t9x3d/1 (accessed on 1 January 2023). BIDMC-PPG-Signals [34]: https://physionet.org/content/bidmc/1.0.0/ (accessed on 2 January 2023). MIMIC-PPG-Signals [35]: https://archive.physionet.org/physiobank/database/mimic3wdb/matched/ (accessed on 2 February 2023).
